# Editorial: The Past and the Future of Human Immunity Under Viral Evolutionary Pressure

**DOI:** 10.3389/fimmu.2019.02340

**Published:** 2019-10-02

**Authors:** Tara Patricia Hurst, Gkikas Magiorkinis

**Affiliations:** ^1^Department of Life Sciences, School of Health Sciences, Birmingham City University, Birmingham, United Kingdom; ^2^Department of Hygiene, Epidemiology and Medical Statistics, Medical School, National and Kapodistrian University of Athens, Athens, Greece

**Keywords:** human viruses, endogenous retrovirus, immunity, evolution, disease

“*Nothing in biology makes sense except in the light of evolution*” was argued by Theodosius Dobzhansky in 1973. After more than 100 years since Charles Darwin's *Origin of Species*, it was increasingly realized that the theory of evolution can help us disentangle biological mechanisms. Here we asked contributors to see their antiviral research interests “through the looking glass” of evolution and to consider how evolutionary theory may help us understand human antiviral immunity. Thus, our special topic explored the scope of the evolution of human antiviral immunity, whether this included short-term effects or long-term impact. We specifically asked contributors to provide thought-provoking manuscripts that could fall within the scope of co-evolution of viruses and the human immune response. They submitted articles discussing the ongoing dynamics of co-existing with ancient endogenous retroviruses, as well as the pressures exerted by extant exogenous viruses such as influenza and human immunodeficiency virus (HIV-1). Our understanding of antiviral immunity was broadened by papers exploring mechanisms such as T cell function and RNA structural components.

In a Hypothesis and Theory article, Marchi et al. explore the effects of persistent infection on T cell responses by analyzing gene expression profiles and gene networks in publicly-available data sets. Importantly, they identified T-box 21 (Tbx21)-mediated gene expression as a hallmark of inflation but not exhaustion of memory CD8+ T cells. This finding has potential to shape our understanding of the genes that define memory T-cell populations. Further, this shows how different viral infections can influence either exhaustion or inflation. This highlights the adaptability of the immune response to viral infection but also how infection could alter gene expression to shift T cell phenotypes.

In one of two mini-reviews in the topic, Smyth et al. discuss the contribution of RNA structure to antiviral immunity. RNA is much more than an intermediary between DNA and protein; it can adopt complex three-dimensional structures that may be altered by single nucleotide mutations, with consequent effects on its functions. Thus, since RNA structures are critical to the replication of many viruses, they are also substrates upon which evolution can act. This can include non-coding RNAs produced during replication, as well as genomic RNA and transcripts. Viral RNA is sensed by different pattern recognition receptors (PRRs) of the innate immune response, such as Toll-like receptor 3 (TLR3) and retinoic acid inducible gene I (RIG-I). There is thus pressure on viral RNA to evade immune detection such as by nucleotide and codon usage bias (adenosine by HIV-1), the presence of specific secondary structures that restrict transcription to low levels, or the maintenance of single-stranded RNA regions to avoid PRR detection. In turn, viral RNA structures have influenced the human immune system, such as the evolution of the human leukocyte antigen (HLA) locus.

The co-evolution of influenza virus and host immunity is discussed in the second mini-review (Voskarides et al.). The authors argue that this is an example of antagonistic evolution, with influenza virus a predator and the human host as prey. This conflict manifests in the genetic variability of Influenza viruses, a result of the absence of proof-reading by the RNA polymerase, that continually challenges the host immune system. In response, the human host has genetic major histocompatibility complex (MHC) diversity which facilitates T cell-mediated immunity to diverse pathogens.

APOBEC3G (apolipoprotein B mRNA editing enzyme catalytic polypeptide-like 3G, A3G) is a cellular cytidine deaminase that mutates retroviral genomes, thereby being a key host cell mechanism to debilitate viruses that embed in the genome. In an original article herein, the authors suggest that the action of A3G extends to adaptive immunity, suggesting that HIV-1 cytotoxic T cell (CTL) escape mutants are generated through A3G activity (Borzooee et al.). The authors use a bioinformatics approach to simulate A3G mutations and show that HIV-1 mutants are biased toward cytotoxic T cell (CTL) escape mutants. These results point toward an evolutionary adaptation of the HIV-1 genome that essentially “hijacks” human antiretroviral activity to counteract T-cell immunity.

A cluster of papers within the topic discuss the role of endogenous retroviruses (ERV) in the immune response. This includes an up-to-date review on the shaping of the immune response by human ERVs (HERVs) (Grandi and Tramontano). Further, four original research articles explore diverse aspects of ERVs and immunity. Firstly, ERV sequences within the genome are a potential source of genetic diversity that are known to be co-opted by the host. This has been shown most recently to include HERV-H sequences contributing to stem cell identity. Gemmell et al. explore the nature of HERV-H loci and their transcription further, showing positive correlations between particular long-terminal repeats (LTRs) and cell types (type II LTRs and embryonic cells).

Secondly, Turnbull and Douville analyzed HERV-K protease sequences within the human genome. They show that transcription of HERV-K proteases varies according to the functional motifs, with two motifs (DTGAD, DTGVD) being more abundant among all HERV-K integrations, but also more active, compared to the less common motifs, in diseases such as amyotrophic lateral sclerosis and breast cancer. These results are important not only for understanding the interactions among HERV-K integrations evolutionarily, but also to appreciate the diversity of enzymatic targets when designing anti-HERV based treatments.

Thirdly, the effect of the histone deactylase (HDAC) inhibitor vorinostat on HERV expression in CD4+ T cells was analyzed using RNA-Seq (White et al.). Vorinostat is one latency reversing agent being examined in HIV-1 shock and kill strategies. The study found over 2000 HERV elements were modulated by vorinostat, notably the downregulation of ERVL and upregulation of HERV-9 elements, the latter of which was confirmed by digital droplet PCR. This study identified three HERV-9 members (LTR12 elements) that were upregulated by vorinostat in a dose-dependent manner. The relevance of this finding lies in the transcriptional regulation of certain genes by LTR12 elements, including the pro-apoptotic genes TP3 and TNFRSF10B (White et al.). In agreement with our previous work using RT-qPCR, members of the HERV-K (HML-2), HERV-W and HERV-FRD families were not found to be upregulated by vorinostat (1). Notably the study by White et al. unlike that of Hurst et al., used uninfected, primary CD4+ T cells to avoid confounding effects of HIV-1 infection.

Finally, ERVs can be upregulated in response to acute exogenous viral infections and this may contribute to the antiviral response. In the paper by Maze et al. the expression of *Papio cynocephalus* Endogenous Retrovirus (PcEV) was found to be upregulated in macaque plasma and tissues (peripheral blood mononuclear cells (PBMCs) and spleen) in response to simian immunodeficiency virus (SIV) infection. Further, upregulation of the interferon (IFN)-stimulated gene (ISG), STAT1, an important marker of the early antiviral response, correlated with the increased PcEV RNA levels (Maze et al.). The authors posit a role for ERVs in the activation of the innate immune response.

The effect of immunity-virus interface at the population level was examined by Williams et al. where they present the perplexing phenomenon of a “pocket” of uninfected individuals in a connected network of recently HIV-1-infected individuals. This raises the question of how a cluster of connected HIV-1 exposed individuals could remain uninfected within an HIV-1 outbreak. The authors discuss potential explanations including the possibility that long-term infections act as a “firewall” in spreading the infections to the full-population. The latter could be explained by the existence of an epidemic equilibrium of co-existence where human-HIV co-evolution would be acting in the absence of treatment.

The evolutionary gameplay underlying the pathogenesis of hepatitis B virus's e antigen (HBVeAg) is reviewed and explored by Kramvis et al. HBVeAg, as the authors explain, is a tolerogen, an antigen that increases the tolerance of HBV infection by the human host. There is significant variability in HBeAg seroconversion and transmission efficiency among genotypes, which the authors suggest can be explained as a result of short-sighted evolution. The virus-host interplay of HBV is further explored by Lumley et al. on the effects of CD8+ T cell responses. The authors review the evidence that CD8+ T cells play an important role in the chronicity of HBV infection as well as the disease outcomes such as cirrhosis and hepatocellular carcinoma (Lumley et al.). The role of interferon-α receptor-1 (IFNAR1) promoter polymorphisms on HBV infection are explored by Karamitros et al. with respect to the development of hepatocellular carcinoma. The authors show that the variable tandem repeat [VNTR: −77(GT)n] has a significant impact in the transcription profile of IFNAR1 and this may be relevant in cancer pathophysiology.

Finally, Beaver et al., have reviewed evidence of how Zika virus (ZIKV) evolution can explain the observed patterns of pathogenesis. The authors review evidence showing that the immune responses against African isolates differ from those observed against Asian ZIKV lineages, and discuss how this could explain observed pathogenic differences among ZIKV strains.

From ancient germline infections to modern epidemics, outbreaks or emerging infections, our contributors explored the evolutionary interplay of human immunity and viruses at the molecular, individual, or population level. They offered thought-provoking hypotheses for further exploration, reviewed publications, and provided novel results in the field of human antiviral immunity field. This shows the power of integrating evolutionary theory with research of antiviral immunity and we hope that our special topic will further promote evolutionary thinking across fields of human pathogen research.

## Author Contributions

TH and GM wrote the draft and edited the final version of the manuscript.

### Conflict of Interest

The authors declare that the research was conducted in the absence of any commercial or financial relationships that could be construed as a potential conflict of interest.

## References

[1] Hurst T Pace M Katzourakis A Phillips R Klenerman P Frater J. Human endogenous retrovirus (HERV) expression is not induced by treatment with the histone deacetylase (HDAC) inhibitors in cellular models of HIV-1 latency. Retrovirology. (2016) 13:10. 10.1186/s12977-016-0242-4 26852322 PMC4744380

